# Efficiently searching through large tACS parameter spaces using closed-loop Bayesian optimization

**DOI:** 10.1016/j.brs.2019.07.003

**Published:** 2019

**Authors:** Romy Lorenz, Laura E. Simmons, Ricardo P. Monti, Joy L. Arthur, Severin Limal, Ilkka Laakso, Robert Leech, Ines R. Violante

**Affiliations:** a MRC Cognition and Brain Sciences Unit, University of Cambridge, Cambridge, CB2 7EF, UK; b Max-Planck Institute for Human Cognitive and Brain Sciences, Leipzig, 04303, Germany; c Computational, Cognitive and Clinical Neuroscience Laboratory, Department of Medicine, Imperial College London, London, W12 0NN, UK; d Gatsby Computational Neuroscience Unit, University College London, London, W1T 4JG, UK; e Department of Physiology, Anatomy and Genetics, University of Oxford, Oxford, OX1 3PT, UK; f Department of Electrical Engineering and Automation, Aalto University, Espoo, 02150, Finland; g Centre for Neuroimaging Science, King's College London, London, SE5 8AF, UK; h School of Psychology, Faculty of Health and Medical Sciences, University of Surrey, Guildford, GU2 7XH, UK

**Keywords:** Transcranial alternating current stimulation, Experimental design, Machine-learning, Bayesian optimization, Real-time, Phosphenes

## Abstract

**Background:**

Selecting optimal stimulation parameters from numerous possibilities is a major obstacle for assessing the efficacy of non-invasive brain stimulation.

**Objective:**

We demonstrate that Bayesian optimization can rapidly search through large parameter spaces and identify subject-level stimulation parameters in real-time.

**Methods:**

To validate the method, Bayesian optimization was employed using participants’ binary judgements about the intensity of phosphenes elicited through tACS.

**Results:**

We demonstrate the efficiency of Bayesian optimization in identifying parameters that maximize phosphene intensity in a short timeframe (5 min for >190 possibilities). Our results replicate frequency-dependent effects across three montages and show phase-dependent effects of phosphene perception. Computational modelling explains that these phase effects result from constructive/destructive interference of the current reaching the retinas. Simulation analyses demonstrate the method's versatility for complex response functions, even when accounting for noisy observations.

**Conclusion:**

Alongside subjective ratings, this method can be used to optimize tACS parameters based on behavioral and neural measures and has the potential to be used for tailoring stimulation protocols to individuals.

## Introduction

A challenge when selecting optimal parameters for studies involving transcranial alternating current stimulation (tACS) is that it rapidly results in a combinatorial explosion of experimental conditions, as physiologically plausible frequencies (0.1–100 Hz) and relative phase differences between electrodes (0–359°) span across a wide range of possibilities; thus, exhaustively testing all possibilities results in impractical experiment durations [1]. To overcome this challenge, active sampling approaches have been developed that automatically choose samples from which they progressively learn in real-time. Active sampling is useful when exploring a large space of possible experimental conditions and when the acquisition of appropriate data comes at a cost, either in terms of time, financial costs, data quality or subject comfort. In psychometrics, the potential of Bayesian active sampling approaches has long been recognized. Typically, parametric Bayesian methods are used [[2], [3], [4], [5]]; however they rely on strong assumptions about the underlying objective function which are difficult to justify given limited a priori knowledge. By contrast, nonparametric Bayesian active sampling provides far more flexibility and allows accommodating many different types of functions. Additionally, they are often faster and conceptually easier to implement [6].

To validate nonparametric Bayesian optimization in the context of non-invasive brain stimulation, we looked at *phosphenes*: illusory flash-like visual percepts that can be reliably induced by tACS. The current understanding is that phosphenes are generated in the retina by current spreading from the stimulation electrodes [[7], [8], [9], [10], [11]]. To date, two tACS-related stimulation parameters have been shown to affect phosphene perception: frequency and intensity. The strongest perception occurs for frequencies in the lower beta range, i.e., 14–22 Hz [12,13], and increases linearly with stimulation intensity [9,12,14]. However, the effect of relative phase has not yet been investigated. Depending on the montage employed surface electrodes can generate superimposition of currents injected at different relative phases, which the retinas might be sensitive to, providing a compelling model to test our nonparametric Bayesian optimization approach.

Thus, for this proof-of-concept, tACS frequency and phase parameters were explored conjointly. Conventionally assessing phosphene perception during tACS involves subjects judging perceived intensity on a rating scale^2^ [9,12,[14], [15], [16], [17]]. This is problematic as it relies on judgments of absolute magnitudes; however, humans are better at making relative judgments [[18], [19], [20], [21], [22]], expressing preference for one option over another. Thus, we applied nonparametric Bayesian optimization [1,23,24] based on relative judgements [25], to search through a large tACS parameter space, with the aim of identifying frequency-phase combinations that elicit the strongest phosphene perception in individual subjects.

## Methods

### Empirical studies

**Fig. 1:**
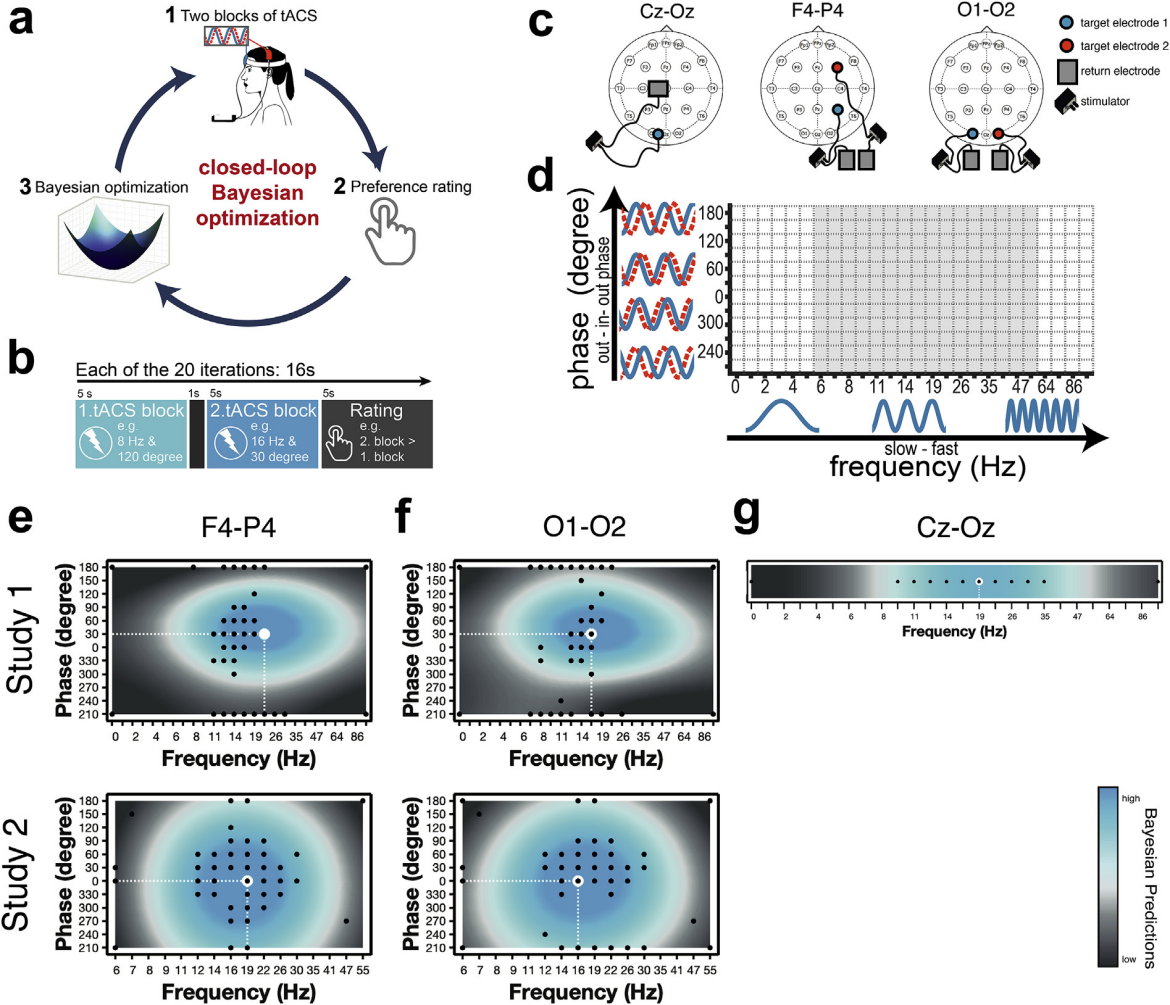
| Methods and empirical group-level results. **(a)** Experimental procedure: (1) Subjects receive two blocks of tACS with different frequency-phase combinations. (2) After both blocks, subjects indicate for which block the phosphene perception was stronger by pressing a button. (3) Based on the subjects' choice, the algorithm automatically proposes a [1] new pair of tACS parameters to be applied in the next iteration. **(b)** Timings per iteration. **(c)** In Study 1, three different montages were tested: Cz-Oz, F4–P4 (connected to separate returns on the right shoulder), and O1–O2 (connected to separate returns on the left and right shoulder). In Study 2, only the latter two were investigated. **(d)** In Study 1, the tACS space searched by Bayesian optimization consisted of 26 (logarithmically scaled) x 12 different frequency-phase combinations (white and grey space). Study 2 zoomed into the space by narrowing the frequency range considered, resulting in 16 × 12 different combinations (grey space). **(e**–**g)** Group-level Bayesian mean models for **(e)** F4–P4, **(f)** O1–O2 and **(g)** Cz-Oz. Blue indicates higher perceived phosphene intensity. Black dots correspond to points sampled by the acquisition function (using a Thurstone-Mosteller model, binary observations from each iteration were related to a single scalar value of the continuous function; many comparisons were identical across subjects resulting in fewer than 20 iterations x 10 subjects dots). The white dashed line indicates the frequency-phase combination with highest perceived phosphene intensity. Subject-level Bayesian mean models are depicted in Supplementary Fig. 2/3. (For interpretation of the references to colour in this figure legend, the reader is referred to the Web version of this article.)

We applied Bayesian optimization (Fig. 1a) in two studies (Study 1: N = 10, 6 females, mean age±SD: 26.3 ± 5.59 years; Study 2: N = 10, 5 females, mean age±SD: 26. 6 ± 4.69 years). All participants reported normal or corrected-to-normal vision. Exclusion criteria were self-reported metal implants in the head or implanted electronic devices, history of neurological problems or head injury, skin sensitivity, pregnancy, use of psychoactive medication. Subjects gave written informed consent for their participation. The study conformed to the Declaration of Helsinki and ethical approval was granted through the local ethics board (NRES Committee London – West London & GTAC).

At each of the 20 iterations, subjects were exposed to two successive blocks of tACS (peak-to-peak amplitude of 1 mA) lasting 5 s each (with 1 s rest between blocks, Fig. 1b). These two blocks differed in the tACS frequency-phase-combination applied to the subject and were selected by the acquisition function (see Bayesian Optimization). Optimization was driven based on the subject's rating of which of the two blocks evoked the strongest phosphene perception. If subjects perceived no clear difference between blocks, or did not perceive phosphenes, they were instructed to select a block at random. Based on the subject's choice, the algorithm automatically proposed a new pair of tACS parameters to be applied in the next iteration (Fig. 1a). In Study 1, three montages were tested in separate runs (within-subject design): Cz-Oz, F4–P4 and O1–O2 (the latter two montages connected to separate returns on the shoulders, see Fig. 1c). Montage Cz-Oz was selected to validate the method against previous studies showing frequency dependency of phosphenes [12]; here phosphene perception was only optimized across frequencies. Montages O1–O2 and F4–P4 were chosen as they generate superimposition of currents that allowed the investigation of the effect of frequency and phase on phosphene perception across different electrode scalp distributions. Results of Study 1 were used to obtain more accurate priors for Study 2. In Study 2, phosphene perception was assessed for montages F4–P4 and O1–O2 at a higher level of detail by narrowing the frequency-range considered (Fig. 1d, grey space).

### Bayesian optimization

Bayesian optimization is a two-stage procedure that repeats iteratively. In the *data modelling stage*, a probabilistic surrogate model is used to estimate the objective function. While commonly, Bayesian optimization requires scalar responses, Brochu et al. [25] have proposed an approach based on preferences, which was employed here (Brochu's Python implementation is available on https://github.com/misterwindupbird/IBO). By using a Thurstone-Mosteller model with a Gaussian process (GP), it is possible to relate binary observations to a continuous function (Supplementary Fig. 4). In the *guided search stage*, an acquisition function is used to propose two points in the parameter space from which to sample next (i.e., chooses the two blocks with different tACS parameters the subject will receive in the next iteration). As such, the acquisition function balances the trade-off between exploring the parameter space and exploiting the parameters for which measurements have already been collected; this allows for an efficient and reliable search over an exhaustive parameter space. Here, we applied the expected improvement (EI) acquisition function (see Supplementary Methods A).

### Computational modelling

Current density on the retinas for montage F4–P4 and O1–O2 was approximated using the finite element method and a realistic head model (see Supplementary Methods B).

### Simulation analyses

Simulations were carried out to demonstrate the method's applicability to variously complex objective functions (featuring multiple local optima), as well as, how the choice of the acquisition function impacts the results (comparing the EI with the upper-confidence bound (GP-UCB) acquisition function). Further, we wanted to understand how instructing participants to give random judgements in cases when they were not able to notice any differences in phosphene intensity, may have affected our results. For each possible combination of human rater sensitivity level, acquisition function and objective function, we ran 50 simulations with the number of iterations set to 39. Identical to the empirical studies, at each iteration, the acquisition function proposes two points in the experiment space to be sampled from. Optimization was driven by the binary information about which of the two points corresponds to a larger “ground truth” function value. For details please refer to the Supplementary Methods C.

## Results

### Replication of frequency-dependent phosphene perception

Group-level Bayesian models were obtained for each study separately by inferring the GP based on all available preference ratings of all subjects for a given montage. Group-level Bayesian mean model for montage Cz-Oz (Fig. 1g; for group-level variance see Supplementary Fig. 1; subject-level results are available in Supplementary Fig. 2) implicate that the strongest phosphene perception was predicted at 19 Hz, in close agreement with previous studies [12].

### Phase-dependent phosphene perception

Group-level Bayesian mean models for montages F4–P4 (Fig. 1e; for group-level variance see Supplementary Fig. 1) show that the strongest phosphene perception was predicted at 22 Hz with 30° (Study 1) and at 19 Hz with 0° (Study 2). For montage O1–O2 (Fig. 1f; for group-level variance see Supplementary Fig. 1), both studies predicted the optimum at 16 Hz, either with 30° (Study 1) or 0° (Study 2) phase difference. Subject-level results are available in Supplementary Fig. 2/3. The log marginal likelihood (lml) of group-level Bayesian models was computed as a function of varying length-scale parameter for “Phase”, while keeping the length-scale for “Frequency” fixed (for details see Supplementary Methods A). Lml automatically incorporates a trade-off between model fit and model complexity, and higher values are desirable. Directly comparing lml for varied “Phase” length-scale parameters, thus serves as a good indicator for assessing phase-dependent effects of phosphene perception. While high lml values for small to medium length-scales (i.e., 1–10) would indicate a phase effect, high lml values for large length-scale parameters (i.e., 20–50) would contradict a phase-dependent effect. Results are listed in Table 1 and indicate a clear effect of phase for both montages as medium-sized length-scale parameters resulted in higher lml values.

**Table 1:** Log marginal likelihood values for different length-scale parameters (1−50) of kernel for the “Phase” dimension (underlined values correspond to maximum log marginal likelihood values for each study).

	1	2	3	4	5	7	10	20	50
Montage F4–P4									
Study 1	−3.95	−0.02	0.64	−1.55	−3.75	−9.65	−33.40	−93.40	−127.88
Study 2	−50.89	−52.06	−37.36	−25.92	−20.87	−20.69	−24.00	−29.49	−31.71
Montage O1–O2									
Study 1	−18.40	−9.93	−6.03	−7.19	−11.15	−25.32	−53.55	106.34	135.21
Study 2	−17.87	−31.62	−27.02	−19.39	−15.85	−14.49	−15.20	−16.86	−17.65

### Computational model explains phase-dependence of phosphenes resulting from wave interference

**Fig. 2:**
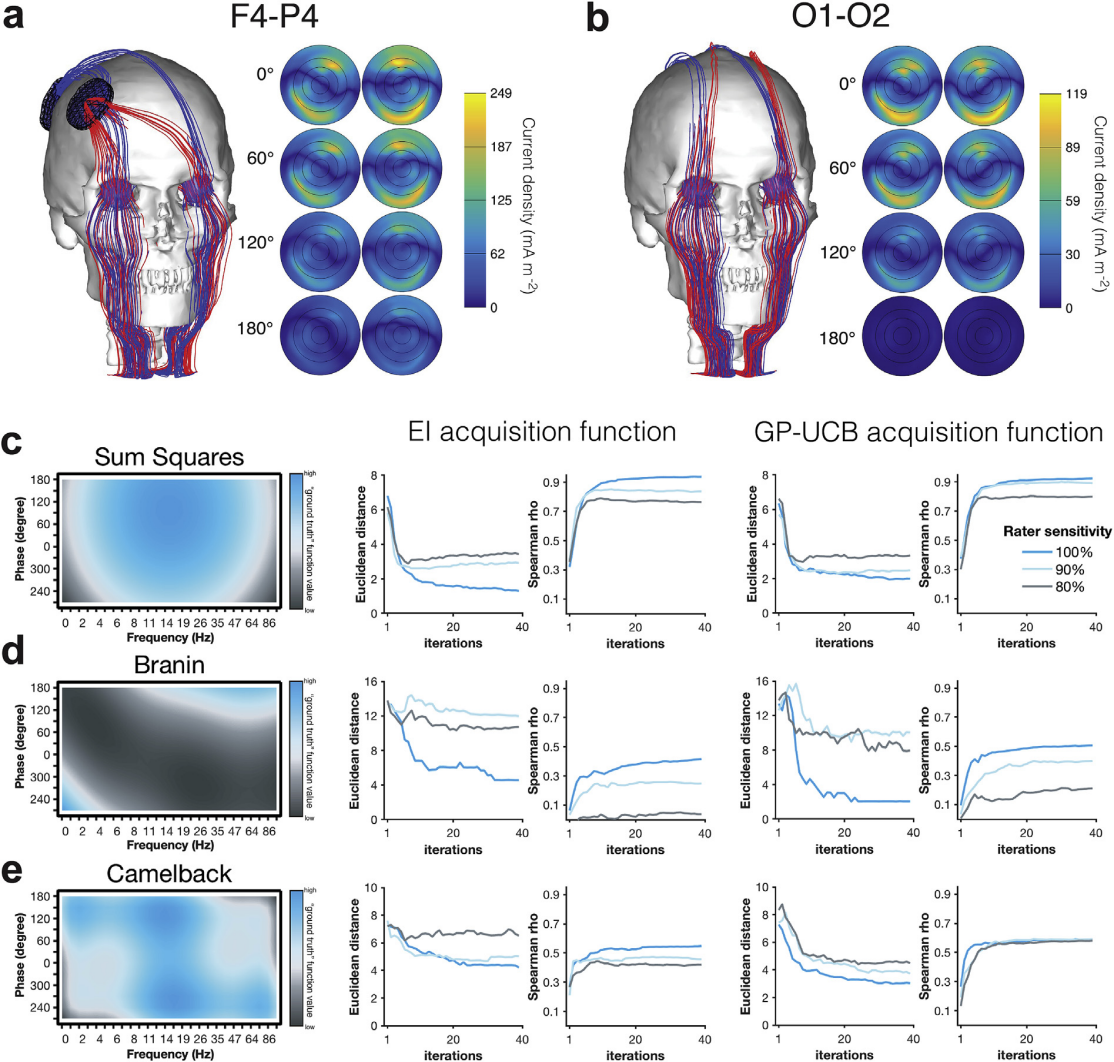
| Results of computational modelling and simulations. **(a**–**b)** Computational modelling (for details see Supplementary Methods B). Left panel: Streamline visualization showing the direction of current for montage **(a)** F4–P4 and **(b)** O1–O2. Only streamlines passing through the eyes are shown. Red and blue streamlines show the currents originating from the two stimulation electrodes. Right panel: The amplitude of the normal component of the current density on left and right retinas (posterior view) for four different phase differences (0°, 60°, 120° and 180°). The component of the current density vector perpendicular to the retina is shown. **(c**–**e)** Simulation analyses for **(c)** Sum Squares, **(d)** Branin and **(e)** Camelback objective functions. For both acquisition functions (EI and GP-UCB), the mean ± SEM (shaded areas) Euclidean distance (across 50 simulations) between predicted and true optimum, and the mean ± SEM (shaded areas) Spearman spatial correlation coefficient between predicted and true objective function were computed for each of the simulated 39 iterations. All simulations were run for three different levels of human rater sensitivity, ranging from 100% (blue) over 90% (light blue) to 80% (grey). (For interpretation of the references to colour in this figure legend, the reader is referred to the Web version of this article.)

According to computational modelling, both F4–P4 (Fig. 2a) and O1–O2 (Fig. 2b) montages yield the highest retinal current density when tACS is applied in phase (i.e., 0°), and the lowest current density when the stimulation is applied at opposite phase (i.e., 180°). In the 0° condition, the currents produced by the two stimulators pass through the eyes with the same phase and approximately in the same direction, producing constructive interference. In the 180° condition, the retinal current densities interfere destructively, and the currents are mostly restricted in between stimulation electrodes.

### Simulation results confirm the versatility of method

When simulating “perfect” human rater sensitivity (i.e., 100%), the algorithm identifies the optimum for all three objective functions, illustrated by the small Euclidean Distance between predicted and true optimum. Equally, the algorithm captures the whole distribution of the functions, reflected in moderate (*rho* > .5) to high spatial correlations (*rho* > .8) between predicted and true functions. For simple (Sum Squares, Fig. 2c) and highly complex functions (Camelback, Fig. 2e), results remain robust even for low human rater sensitivity levels. This is not the case for the moderately complex function (Branin, Fig. 2d), for which the algorithm seems to settle for the local optimum instead. In this case but also more generally, we observe the explorative GP-UCB acquisition function resulting in better results than the EI acquisition function especially for low human rater sensitivity levels.

## Discussion

We demonstrate that Bayesian optimization based on binary preference ratings provides a feasible and efficient method for searching through large tACS parameter spaces (312/192 possible combinations in Study 1/Study 2). While hypothetically testing each possible parameter combination would have taken up to 83/51 min in Study 1/Study 2, the method took 5 min per participant. We not only replicate well-known frequency-dependent effects, but we also demonstrate phase-dependent interference effects of phosphene perception. Phase dependency is consistent with the changes of the current density on the retinas, which supports the hypothesis of retinal origin of tACS-induced phosphenes [[7], [8], [9], [10], [11]]. This effect is relatively subtle when compared to frequency-dependent effects; therefore, more conventional rating scales may have lacked sufficient sensitivity to assess these differences in human perception.

While we show optimization based on subjective ratings, importantly this approach can be used to find the optimal tACS parameters for behavioral (e.g., reaction time) or neural (e.g., fMRI or EEG) target measures [23,24,[26], [27], [28]]); in those cases it can also optimize scalar values instead of binary observations [25]. Our simulation analyses showed that the method can model variously complex and noisy objective functions. This is critical as we hypothesize that identifying the optimal tACS frequency-phase combination for effectively modulating cognitive processes might be more challenging: the relationship between tACS parameters and the subject's neural or behavioral response could be less uniform, feature multiple optima and may be affected by a lower contrast-to-noise ratio. Interestingly, the approach can also be extended to search through multi-dimensional parameter spaces (e.g., including stimulation intensity as an additional dimension). While this approach has originally been proposed in robotics, artificial intelligence [29] and computer graphics [25], this study may pave the way to an application in the field of brain stimulation. As the method works on a per subject-basis, it has the potential to be used for tailoring stimulation protocols to individuals.

## Footnotes

2 The standard procedure to assess tACS-induced phosphene perception is to expose participants to the theoretically assumed strongest tACS frequency (such as 16 Hz) before the experiment starts, thereby acting as a “reference” for subsequent rating blocks. However, this requires for subjects to recall the intensity of the reference phosphene intensity while being exposed to blocks of other frequencies. Thus, this procedure is less precise for determining subtle differences in phosphene perception.
