# Supplementary material for: Efficiently searching through large tACS parameter spaces using closed-loop Bayesian optimization
Source: Brain Stimul. 2019 Nov-Dec;12(6):1484–9. doi: 10.1016/j.brs.2019.07.003 (PMC6879005; doi:10.1016/j.brs.2019.07.003)
Supplement: Multimedia component 1 [file mmc1.pdf]

Supplementary Material

Efficiently searching through large tACS parameter spaces using closed-loop Bayesian optimization

Romy Lorenz, Laura E. Simmons*, Ricardo P. Monti*, Joy L. Arthur, Severin Limal, Ilkka Laakso, Robert Leech and Ines R. Violante

*These authors contributed equally to this work.

Overview

Supplementary Figure 1	Group-level Bayesian variance	p. 2
Supplementary Figure 2	Subject-level Bayesian models of Study 1	p. 3
Supplementary Figure 3	Subject-level Bayesian models of Study 2	p. 4
Supplementary Figure 4	GP regression based on binary observations	p. 5

Supplementary Methods A	Empirical studies	p. 6 - 9
Supplementary Methods B	Computational modelling	p. 10
Supplementary Methods C	Simulation analyses	p. 10-11

Supplementary Table 1	Side effects of stimulation	p. 12

Supplementary References	p. 13

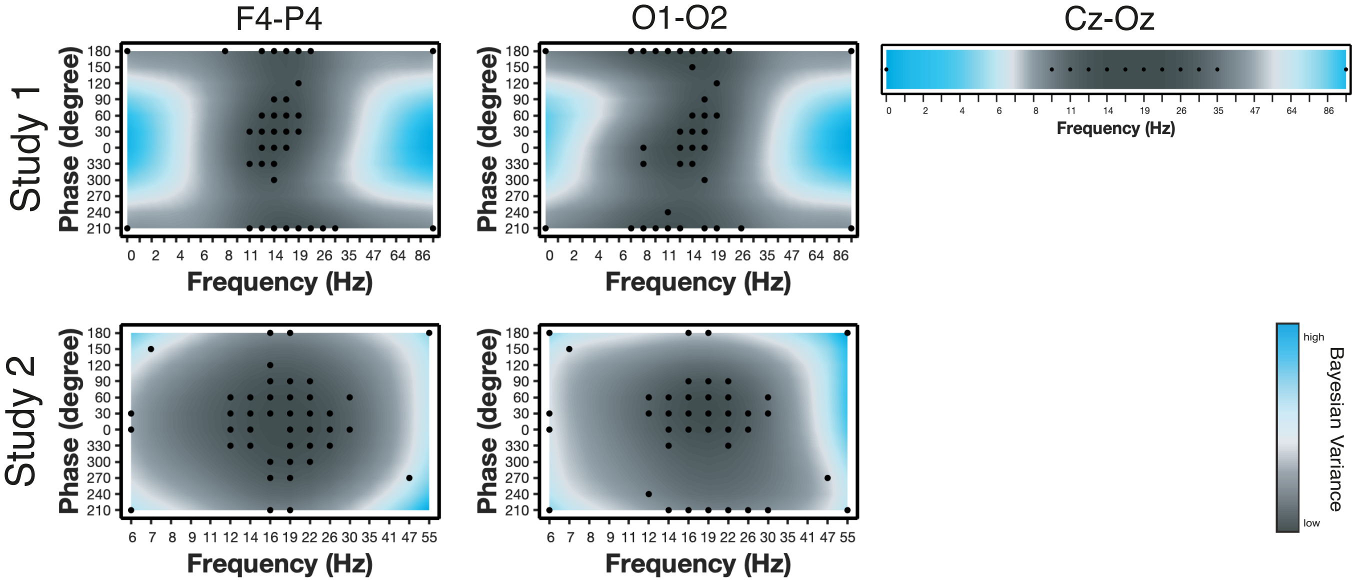

Supplementary Figure 1: Group-level Bayesian variance for montages F4-P4, O1-O2 and Cz-Oz. Blue indicates higher variance. Black dots correspond to points sampled by the acquisition function (using a Thurstone-Mosteller model, binary observations from each iteration were related to a single scalar value of the continuous function; many comparisons were identical across subjects resulting in fewer than 20 iterations x 10 subjects dots, see Supplementary Figure 2/3).

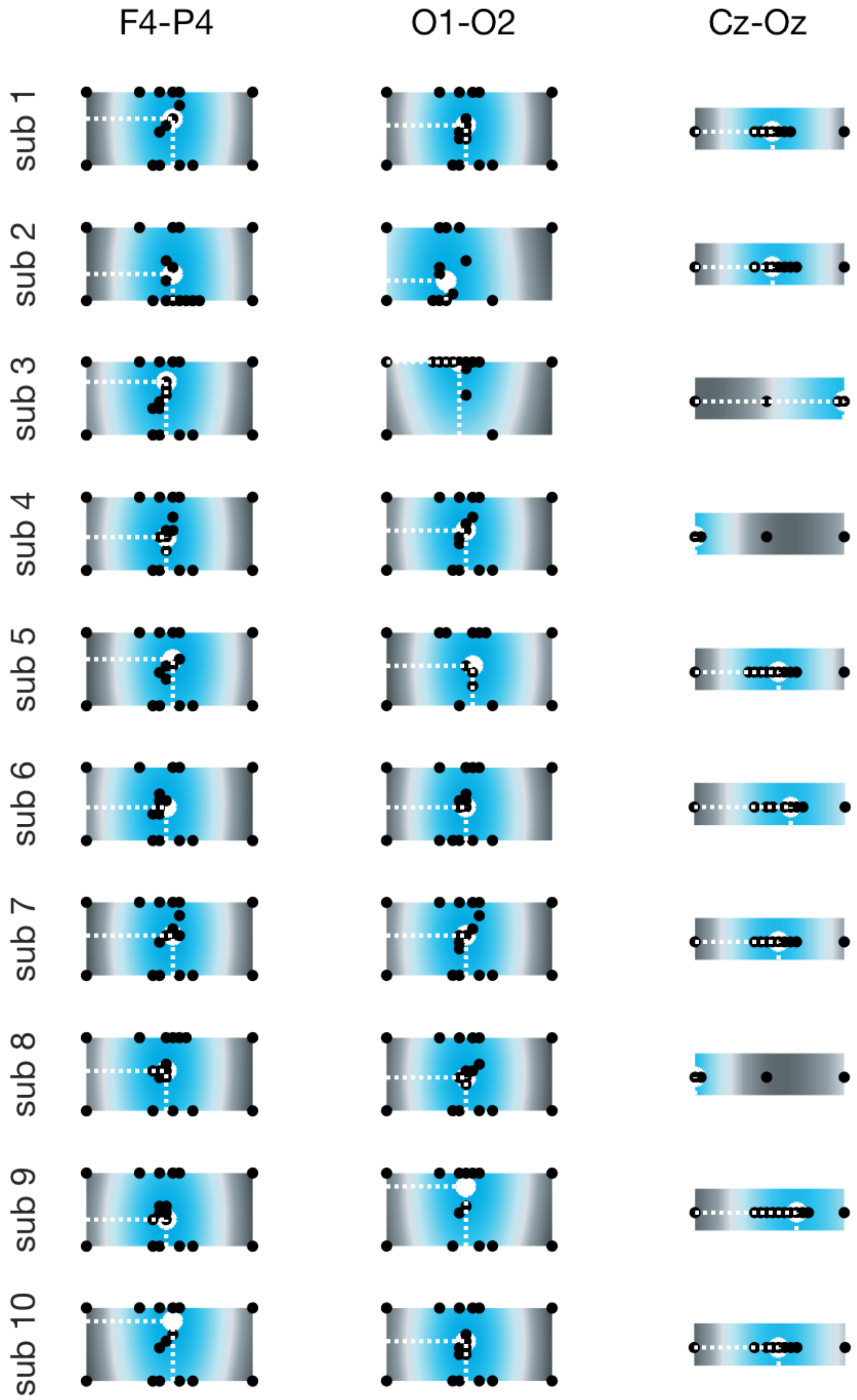

Supplementary Figure 2: Subject-level Bayesian models of Study 1. Bayesian predictions are based on all available observations from a single run with fixed hyper-parameters (same as in real-time setting). Blue indicates higher perceived phosphene intensity. Black dots correspond to points sampled by the acquisition function (using a Thurstone-Mosteller model, binary observations from each iteration were related to a single scalar value of the continuous function; some comparisons were identical in the same run resulting sometimes in fewer than 20 dots). The white dashed line indicates the tACS frequency-phase combination with the highest perceived phosphene intensity. Subjects 3 4, 8 and 9 did report no phosphene perception for montage Cz-Oz and were excluded from subsequent analyses related to this specific montage. Group-level results of Study 1 are depicted in Figure 1e-g in main text (top row).

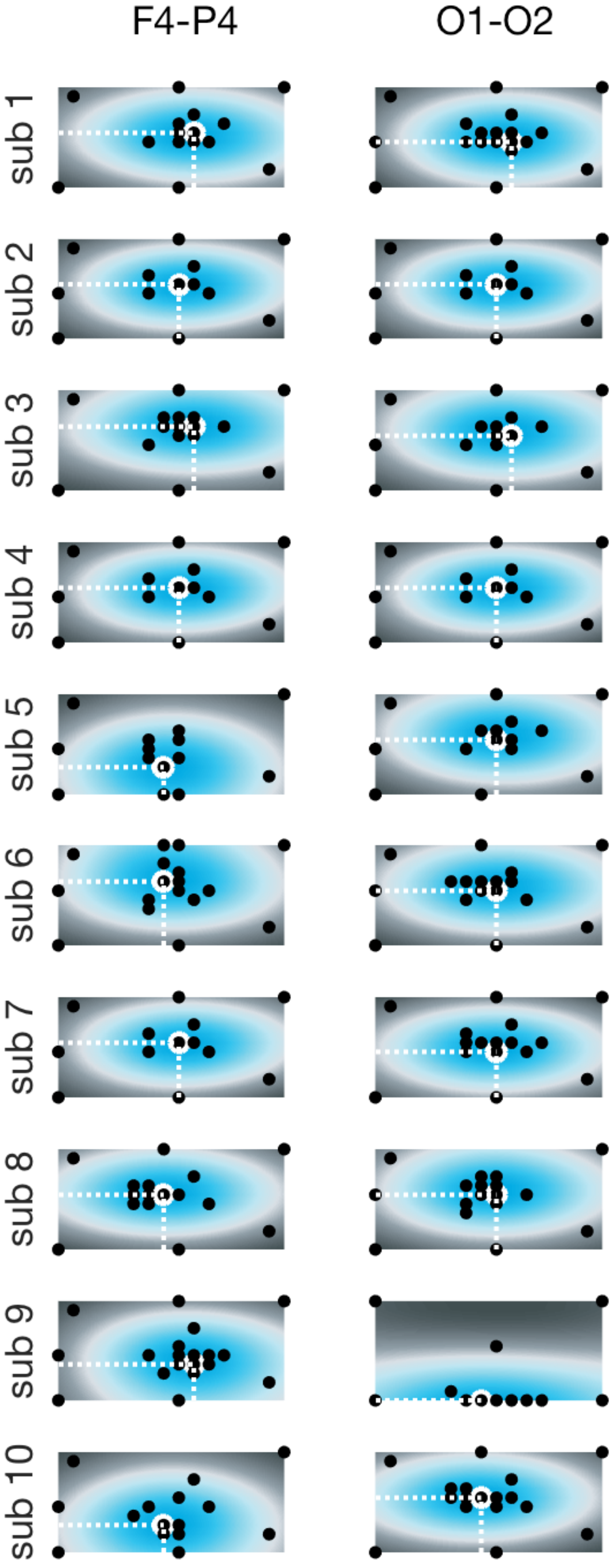

Supplementary Figure 3: Subject-level Bayesian models of Study 2. Bayesian predictions are based on all available observations from a single run with fixed hyper-parameters (same as in real-time setting). Blue indicates higher perceived phosphene intensity. Black dots correspond to points sampled by the acquisition function (using a Thurstone-Mosteller model, binary observations from each iteration were related to a single scalar value of the continuous function; some comparisons were identical in the same run resulting sometimes in fewer than 20 dots). The white dashed line indicates the tACS frequency-phase combination with the highest perceived phosphene intensity. Group-level results of Study 2 are depicted in Figure 1e-g in main text (bottom row).

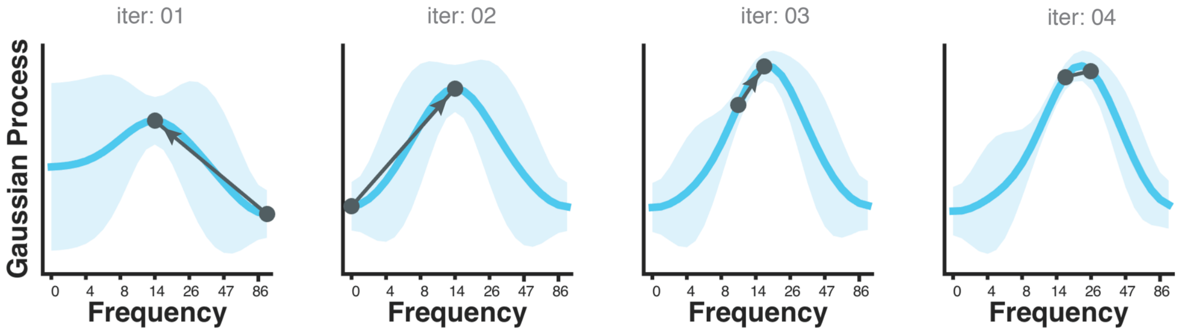

Supplementary Figure 4: GP regression based on binary observations. At each iteration ("iter"), subjects indicate their preference between two points from the parameter space (i.e., the direction of the arrow represents preference). This preference serves as the input to a function that models a GP taking into account all preference ratings given up to that point, as well as prior assumptions about the smoothness of the function. It can be seen, that uncertainty (i.e., variance around the mean) about the predictions decreases over time as more preference ratings are available.

Supplementary Methods A: Empirical studies

TACS montage

Stimulation was delivered using two battery-driven stimulators (NeuroConn GmbH, Ilmenau, Germany) and controlled using Matlab (Mathworks, Natick, MA, USA) via a data acquisition device (NI USB-6343, National Instruments, Newbury, UK). Stimulation was sinusoidal, with peak-to-peak amplitude of 1 mA, and no DC offset. The magnitude of the stimulation applied was the same across scalp electrodes. For both studies, a within-subject design was applied, testing phosphene perception with different montages in separate runs for each participant. A balanced Latin square design was used to alternate the order in which subjects were tested with each montage. For the different montages, conductive rubber electrodes were positioned on the scalp based on locations according to the International 10-20 EEG system (Klem et al. 1999). For Study 1, three different montages were tested (Figure 1c in main text): Cz-Oz, F4-P4 and O1-O2. For the O1-O2 montage, return electrodes were placed on the left and right shoulders, respectively. For F4-P4, both return electrodes were placed ipsilaterally on the right shoulder. For both these montages, each stimulator was connected to a target scalp and its respective return electrode. The Cz-Oz montage used only two electrodes, with the larger return electrode at the Cz position (for this montage stimulation was delivered using one stimulator). Impedances were kept below 10 kΩ using a conductive paste (Ten20, D.O. Weaver, Aurora, CO, USA), which also held the electrodes in place. The target stimulation electrodes were round (diameter = 4.3 cm, thickness: 1mm) while return electrodes were of a rectangular shape (7.3 x 5.4 cm, thickness: 2 mm). Between montages, alcohol wipes were used to remove residue of the conductive paste from the scalp.

TACS parameter space

In Study 1, montage Cz-Oz was optimized for a 1D parameter space (26x1) consisting of logarithmically spaced frequencies ranging from 0 to 100 Hz. Logarithmic scaling was selected due to the convention in the electroencephalogram (EEG) literature where frequencies are often conceptualized on a logarithmic scale (Cohen 2014). For the two other montages, an exhaustive 2D parameter space (26x12) was designed, adding a further dimension consisting of relative phase differences ranging from 0-330° (Figure 1d in main text). Based on the results of Study 1, Study 2 zoomed into the parameter space by narrowing the frequency range considered (6 – 55 Hz, grey shaded area in Figure 1d in main text).

Experimental Procedure

Subjects were seated centrally in front of a computer screen at a distance of 75 cm. The lights in the room were kept on throughout. For each of three electrode montages, subjects were habituated to the stimulation with the intensity increasing incrementally (0.25 mA, 0.5 mA and 1 mA). The first run was preceded by 5 s of stimulation at 16 Hz (0°); it has been reported that the majority of individuals perceives phosphenes at this frequency (Kanai et al. 2008), therefore this served to demonstrate to subjects what phosphene perception “looks” like.

Each run consisted of 20 rating blocks. During the stimulation, subjects were asked to fixate on the words “1.block” or “2.block” as they appeared on the screen (white font on grey background); during the rest period, they were asked to fixate on a white cross in the center of the screen (grey background). After each pair of blocks, subjects were prompted to select the block in which they felt their perception of phosphenes had been stronger. The concept of “stronger” perception was explained before the experiment as: the phosphenes appearing brighter; the phosphenes taking over a larger area of the visual field; phosphenes appearing in both eyes where previously they had only appeared in one; or phosphenes appearing to be more intense or flickering more rapidly. Subjects made their choice between block 1 and 2 by pressing either the left or right button on a button response box.

After each run, participants completed a questionnaire to assess possible side-effects of the tACS stimulation by rating from 0 (none) to 4 (severe) the intensity and duration of: pain, burning, warmth/heat, itchiness, pitching, metallic taste, fatigue, effect on performance or any other side-effect perceived, in accordance with Brunoni et al. (2011). Results of side effects of stimulation are reported in Supplementary Table 1.

Phosphene visibility

The experimenter noted when subjects reported that they did not experience any phosphenes during the entire run. For montage Cz-Oz in Study 1, four subjects reported that they did not perceive any phosphenes over the course of the whole run (see Supplementary Figure 2). These subjects were excluded from subsequent analyses related to this specific montage. For the two other montages, all subjects reported phosphenes in both studies.

Bayesian optimization based on preference ratings

Data modelling stage: In order to estimate a Gaussian process (GP) model relating to preference data, we followed an approach proposed by Brochu et al. (Brochu, Cora, and de Freitas 2010). From a methodological perspective, this approach treats preferential data provided by subjects as a classification task. A GP is employed to model the underlying objective function associated with each input. This surrogate model serves to capture the preference for each input such that if input i_1 is preferred to input i_2 the surrogate function at i_1 should be greater than at i_2. In the study presented here, the distinct inputs correspond to two distinct tACS frequency-phase combinations.

Formally, for a candidate pair of inputs, i_1 and i_2, the associated surrogate function is modelled as follows:

1.

$$\mathrm{v}(\mathrm{i}_1) = f(\mathrm{i}_1) + \varepsilon$$
$$\mathrm{v}(\mathrm{i}_2) = f(\mathrm{i}_2) + \varepsilon$$

where GP prior to $f(\,)$ is assigned. Following Brochu et al. (Brochu, Cora, and de Freitas 2010), the difference in the surrogate function $f(\mathrm{i}_1) - f(\mathrm{i}_2)$ is studied in order to predict whether input i_1 will be preferred. Formally, a probit link function is employed in order to obtain a probability that i_1 is preferred to i_2 as follows:

2.

$$P(\mathrm{i}_1 > \mathrm{i}_2) = \mathrm{q}(\mathrm{z})$$

where $\mathrm{q}(\,)$ is the cumulative distribution function of a standard normal random variable and z is defined as:

3.

$$\mathrm{z} = \frac{f(\mathrm{i}_1) - f(\mathrm{i}_2)}{\sqrt{2}\,\sigma_{\mathrm{noise}}}$$

Given a set of training data, fitting such a model corresponds to fitting a GP for a classification task (Bishop 2007). Due to the non-linear nature of the probit link function an analytic solution is not available for the surrogate function. While a variety of methods have been proposed, here a Laplace approximation was employed as proposed by Brochu et al. (Brochu, Cora, and de Freitas 2010); for further details see (Bishop 2007). The idea of employing a surrogate function to model binary observations has been employed extensively within the statistics and machine learning literature. When a probit link function is used, as in the present work, such an approach is referred to as Thurstone-Mosteller model.

GPs are fully specified by their mean and covariance functions. As a prior, we employed a zero mean function and as covariance function we chose an anisotropic squared exponential (SE) kernel (Rasmussen and Williams 2006). The SE kernel encodes the basic prior assumption

that points close in the task space elicit similar responses while points far from each other could exhibit distinct responses:

4.

$$k(x_i, x_j) = \sigma^2 \exp\left\{-\frac{(x_i - x_j)^2}{2\, l^2}\right\}$$

where x_i and x_j each correspond to a tACS frequency-phase combination, and $(x_i - x_j)^2$ determines their Euclidean distance in the parameter space. The parameters σ^2 and l each determine the variance and length scale of the covariance kernel, respectively; they are referred to as hyper-parameters of the covariance kernel. For Study 1, length-scale parameters of the SE kernel were tuned based on a prior offline study, assessing preference ratings across a smaller parameter space (10 frequencies x 6 phases) with eleven participants for montage F4-P4, and five participants for montage O1-O2. Based on the data from Study 1, length-scale parameters were retuned for Study 2. In both cases, hyper-parameters were selected using type II maximum likelihood (Rasmussen and Williams 2006), implemented via a grid search algorithm (see section "Phase-dependency of phosphene perception" for details). While length-scale parameters differed across studies, the same length-scale parameters were employed for different montages within each study and were kept fixed for all subjects.

Guided search stage: In the context of preferential data the acquisition function must effectively select two points so that a comparison is possible. Brochu et al. (Brochu, Cora, and de Freitas 2010) describe an approach where the first point proposed always corresponds to the current maximum $f(x^+)$, while the second point is then selected by maximizing the expected improvement. The expected improvement (EI) is defined as:

5.

$$EI(x) = \mu(x) - f(x^+)q(z) + \sigma(x)p(z),$$

where $p(\,)$ is the probability density function of a standard normal random variable, $q(\,)$ is the cumulative distribution function of a standard normal random variable and z is defined as:

6.

$$z = \frac{\mu(x) - f(x^+)}{\sigma(x)},$$

for which $\mu(x)$ and $\sigma(x)$ are the mean and standard deviation of the Gaussian predictive posterior distribution at point x. Since the underlying surrogate function follows a GP, this type of acquisition function can be used to select new candidate points in the parameter space. Informally, this choice of acquisition can be seen as trying to maximize the expected improvement over the current best. Brochu et al. (Brochu, Cora, and de Freitas 2010) ran an extensive empirical study and found that the EI acquisition function was preferable to a random acquisition or an acquisition based on the point of highest uncertainty.

The initial pair of tACS parameters proposed by the acquisition function was identical across subjects as a set of points was chosen that maximized variance given the prior (Brochu, Cora, and de Freitas 2010). For proposing points in the parameter space, the acquisition function returned fractions instead of integers; therefore, fractions were rounded to obtain desired integer points, corresponding to a pre-defined tACS frequency-phase combination from the parameter space. However, in some rare cases this procedure resulted in the acquisition function proposing the same tACS frequency-phase combination for comparison at a given iteration (especially towards end of the run for Study 2, when the acquisition function gained more and more certainty about the optimum). As this was unintended by the experimenters, those iterations were removed from post-hoc analyses; in total this resulted in removing 6 out of 400 iterations in Study 1 (F4P4: 5/200; O1-O2: 1/200), and 47 out of 400 iterations from Study 2 (F4P4: 31/200; O1-O2: 16/200). The removal of these iterations was motivated by the fact that corresponding preference ratings were random and therefore introduced undesirable variance into the GP models. For the simulation analyses we circumvented this issue, by

simply using the best and second best point proposed by the acquisition function for such cases.

Phase-dependency of phosphene perception

As mentioned above, an anisotropic covariance kernel was employed in both studies with two independent kernel length-scale parameters for the two dimensions “Frequency” and “Phase”. To assess if the data from the two studies indicated an effect of phase on phosphene perception, the type II maximum likelihood of the group-level Bayesian models was computed as a function of varying length-scale parameter for “Phase” (i.e. 1,2,3,4,5,7,10,20, and 50), while keeping the length-scale parameter for “Frequency” fixed. The type II maximum likelihood estimation corresponds to a computationally feasible approximation where the log marginal likelihood is maximized with respect to the hyper-parameters (Rasmussen and Williams 2006):

7.

$$\log P(\mathrm{y}|\mathrm{X},\theta) = -\frac{1}{2}\mathrm{y}^{\mathrm{T}}\,\mathbf{K}^{-1}\mathrm{y} - \log|\mathbf{K}| - \frac{\mathrm{n}}{2}\log 2\pi$$

where

$$\mathbf{K} = \begin{bmatrix} \mathrm{k}(\mathrm{x}_1,\mathrm{x}_1) & \cdots & \mathrm{k}(\mathrm{x}_1,\mathrm{x}_\mathrm{t}) \\ \vdots & \ddots & \vdots \\ \mathrm{k}(\mathrm{x}_\mathrm{t},\mathrm{x}_1) & \cdots & \mathrm{k}(\mathrm{x}_\mathrm{t},\mathrm{x}_\mathrm{t}) \end{bmatrix}$$

describes the kernel matrix, with k being the anisotropic squared exponential (SE) kernel as defined above in equation 4, n corresponds to the number of observations, and y corresponds to the measured response (i.e., a scalar derived from the Thurstone-Mosteller model as detailed above (Data modelling stage)).

From this equation, it can be seen that the log marginal likelihood is conditioned on the parameters of the kernel θ. The equation consists of three easily interpretable terms:

(1) $-\frac{1}{2}\mathrm{y}^{\mathrm{T}}\,\mathbf{K}^{-1}\mathrm{y}$ is the data-fit
(2) $\log|\mathbf{K}|$ is the complexity penalty
(3) $\frac{\mathrm{n}}{2}\log 2\pi$ is a constant term

While the data fit decreases monotonically with the length-scale as the GP model become less flexible, the penalty term decreases with the length-scale as the GP model becomes less complex. Therefore, the log marginal likelihood automatically incorporates a trade-off between model fit and model complexity (as it takes the data fit minus the penalty term), and higher values are desirable.

Directly comparing log marginal likelihood for varied “Phase” length-scale parameters, thus serves as a good indicator for assessing phase-dependent effects of phosphene perception. While high log marginal likelihood values for small to medium length-scales (i.e., 1-10) would indicate a phase effect, high log marginal likelihood values for large length-scale parameters (i.e., 20-50) would contradict a phase-dependent effect, as such smooth kernels would result in similar predictions along the “Phase”-axis.

Supplementary Methods B: Computational Modelling

Current density on the retinas for each electrode montage was approximated computationally using an anatomically realistic volume conductor model of a 34 year-old male (Christ et al. 2010). The details of the computational procedure, including the tissue electrical conductivities, have been reported previously in Laakso and Hirata (2013). Briefly, the electric scalar potential was determined using the finite-element method with first-order cubical elements (0.5 mm side length), and the current density was calculated by multiplying the gradient of the scalar potential by the tissue conductivity. The frequency of stimulation was assumed to have a negligible effect on the tissue conductivities, and thus, the calculated current density was independent of frequency. The electrodes were modelled as rubber pads (0.1 S/m), the size and thickness of which were identical to the electrodes used in the experiments. A 2.5 mm thick layer of conductive electrode paste (1.6 S/m) was added between the electrodes and the scalp. Return electrodes were modelled using the Neumann boundary condition in the neck. Identically to the experiments, the current intensity was 1 mA.

The effect of phase was modelled by running two computer simulations for each electrode montage. In each simulation, only one pair of electrodes was active, and the other pair was modelled as passive conductors. The total current for an arbitrary phase difference was calculated as a linear combination of the currents of the electrode pairs (Dmochowski et al. 2011). As the eye is mainly sensitive to currents perpendicular to its surface (Brindley 1955), we reported the normal component of the retinal current density.

Supplementary Methods C: Simulation Analyses

As "ground truth" objective functions, we selected three commonly used objective functions: Sum Squares, Branin and Camelback (Molga and Smutnicki 2005; Picheny, Wagner, and Ginsbourger 2013). To make our simulation results comparable to our empirical results, we restricted the search space to consist of two-dimensional integer values of the same dimension as in Study 1 (i.e., 26 x 12 different tACS frequency-phase combinations). In addition to considering the acquisition function used during actual data collection (the expected improvement (EI) acquisition function), we also studied the sampling behavior of another acquisition function: the upper-confidence bound (GP-UCB) (Brochu, Cora, and de Freitas 2010). While the GP-UCB also favors the selection of points with high mean values (similar to the EI acquisition function), it also favors points with high variance (i.e., regions worth to explore). In certain settings, this will result in more explorative behavior for the GP-UCB compared to the more "greedy" EI acquisition function.

To understand how the instruction to provide random judgements for non-perceivable differences in phosphene perception could have affected our results, we simulated three different levels of "human rater sensitivity". For this, we first took the difference between the maximum value of the objective function (i.e., optimum) and all other values, resulting in 312 difference values (i.e., 26 x 12), which were subsequently sorted in descending order and linearly interpolated to generate 1000 difference values. Next, we selected three different "human rater sensitivity" threshold levels: 100%, 90% and 80% corresponding to the 1000th (i.e., smallest difference), the 900th and 800th difference value of the interpolated and sorted data, respectively. The rationale of this approach was to simulate "perfect" sensitivity for even the smallest differences in phosphene intensity (i.e.,100%) and more realistic human rater sensitivity of 90% or 80%. For each possible combinations of human rater sensitivity level (x3), acquisition function (x2) and objective function (x3), we ran 50 simulations, resulting in 900 simulations in total.

Identical to the empirical experiments, in the simulation analyses, at each iteration, the acquisition function proposes two points (i_1 and i_2) in the experiment space to be "presented to the subject" (i.e., a new pair of tACS parameters). As described above in the section "Bayesian optimization based on preference ratings", the information entered into the algorithm

is the relative judgement of which of these two points elicits “stronger phosphenes” which corresponds to a larger “ground truth” objective function value in the simulations (i.e., input into the algorithm is either i_1 > i_2 or i_1 < i_2). When the absolute difference between the objective function values of these two proposed points was below the sensitivity threshold level for the given simulation, the relative judgment entered into the algorithm was set to be random; for all other cases the algorithm was fed with the “ground truth” relative judgment.” In this way, we recreated experimental conditions identical to those in our empirical study, but with a known ground truth underlying objective function. The maximum number of iterations for each simulation run was set to 39.

For each objective function, before any closed-loop simulations were ran, 50 randomly selected observations across the experiment space (i.e., samples of the objective function) were used to identify the optimal covariance kernel (isotropic vs. anisotropic squared exponential kernel vs. Matern kernel) and hyper-parameters using type II maximum likelihood (Rasmussen and Williams 2006), implemented via a grid search algorithm.

As mentioned in the section “Guided search stage”, for some cases in our empirical study, the acquisition function proposed two identical points in the experiment space to be assessed at a given iteration. This was due to rounding values returned by the acquisition function into integers. As such comparisons can be non-informative and add undesirable variance into the GP models; in our simulation analyses, we circumvent this issue, by simply using the best and next best point proposed by the acquisition function for such cases.

Supplementary Table 1: Side effects of stimulation

Summary statistics for adverse effects questionnaire (Brunoni et al. 2011) for all three montages tested across Study 1 and Study 2. Subjects rated each item between 1 (absent) and 4 (severe).

	Montage O1-O2			Montage F4-P4			Montage Cz-Oz		
	Average intensity (SD)	Number of incidences > 1 (out of 20)	Range	Average Intensity (SD)	Number of incidences > 1 (out of 20)	Range	Average Intensity (SD)	Number of incidences > 1 (out of 10)	Range
**Headache**	1.11 (0.32)	2	1-2	1.16 (0.37)	3	1-2	1 (0)	0	1
**Neck Pain**	1.21 (0.54)	3	1-3	1.16 (0.37)	3	1-2	1.10 (0.32)	1	1-2
**Scalp Pain**	1.16 (0.37)	3	1-2	1.47 (0.61)	8	1-3	1.20 (0.42)	2	1-2
**Burning**	1.37 (0.60)	6	1-3	1.58 (0.90)	7	1-4	1.30 (0.48)	3	1-2
**Warmth/heat**	1.37 (0.50)	7	1-2	1.42 (0.61)	7	1-3	1.60 (0.52)	6	1-2
**Skin redness/flush**	1.10 (0.32)	2	1-2	1.05 (0.23)	1	1-2	1 (0)	0	1
**Tingling**	2.16 (0.69)	16	1-3	2.16 (0.50)	18	1-3	1.90 (0.32)	9	1-2
**Pinching**	1.37 (0.60)	6	1-3	1.42 (0.61)	7	1-3	1.50 (0.85)	3	1-3
**Itching**	1.68 (0.89)	9	1-4	1.31 (0.48)	6	1-2	1.10 (0.32)	1	1-2
**Metallic taste**	1.37 (0.76)	4	1-3	1.47 (0.84)	5	1-3	1.10 (0.32)	1	1-2
**Sleepiness/Fatigue**	1.48 (0.77)	6	1-3	1.42 (0.77)	5	1-3	1.30 (0.48)	3	1-2
**Trouble concentrating**	1.32 (0.58)	5	1-3	1.13 (0.45)	5	1-2	1.10 (0.32)	1	1-2
**Effect on performance**	1.16 (0.50)	2	1-3	1.16 (0.37)	3	1-2	1 (0)	0	1
**Acute mood change**	1 (0)	0	1	1	0	1	1 (0)	0	1

Supplementary References

Bishop, Christopher. 2007. *Pattern Recognition and Machine Learning*. New York: Springer.

Brindley, G. S. 1955. "The Site of Electrical Excitation of the Human Eye." *The Journal of Physiology* 127 (1): 189–200.

Brochu, Eric, Vlad M. Cora, and Nando de Freitas. 2010. "A Tutorial on Bayesian Optimization of Expensive Cost Functions, with Application to Active User Modeling and Hierarchical Reinforcement Learning," December. https://doi.org/arXiv: 1012.2599.

Brunoni, Andre Russowsky, Joao Amadera, Bruna Berbel, Magdalena Sarah Volz, Brenno Gomes Rizzerio, and Felipe Fregni. 2011. "A Systematic Review on Reporting and Assessment of Adverse Effects Associated with Transcranial Direct Current Stimulation." *The International Journal of Neuropsychopharmacology* 14 (8): 1133–45. https://doi.org/10.1017/S1461145710001690.

Christ, Andreas, Wolfgang Kainz, Eckhart G. Hahn, Katharina Honegger, Marcel Zefferer, Esra Neufeld, Wolfgang Rascher, et al. 2010. "The Virtual Family--Development of Surface-Based Anatomical Models of Two Adults and Two Children for Dosimetric Simulations." *Physics in Medicine and Biology* 55 (2): N23-38. https://doi.org/10.1088/0031-9155/55/2/N01.

Cohen, Mike X. 2014. *Analyzing Neural Time Series Data: Theory and Practice*. Issues in Clinical and Cognitive Neuropsychology. Cambridge, Massachusetts London: MIT Press.

Dmochowski, Jacek P., Abhishek Datta, Marom Bikson, Yuzhuo Su, and Lucas C. Parra. 2011. "Optimized Multi-Electrode Stimulation Increases Focality and Intensity at Target." *Journal of Neural Engineering* 8 (4): 046011. https://doi.org/10.1088/1741-2560/8/4/046011.

Kanai, Ryota, Leila Chaieb, Andrea Antal, Vincent Walsh, and Walter Paulus. 2008. "Frequency-Dependent Electrical Stimulation of the Visual Cortex." *Current Biology* 18 (23): 1839–43. https://doi.org/10.1016/j.cub.2008.10.027.

Klem, G. H., H. O. Lüders, H. H. Jasper, and C. Elger. 1999. "The Ten-Twenty Electrode System of the International Federation. The International Federation of Clinical Neurophysiology." *Electroencephalography and Clinical Neurophysiology. Supplement* 52: 3–6.

Laakso, Ilkka, and Akimasa Hirata. 2013. "Computational Analysis Shows Why Transcranial Alternating Current Stimulation Induces Retinal Phosphenes." *Journal of Neural Engineering* 10 (4): 046009. https://doi.org/10.1088/1741-2560/10/4/046009.

Molga, Marcin, and Czesław Smutnicki. 2005. "Test Functions for Optimization Needs," 43.

Picheny, Victor, Tobias Wagner, and David Ginsbourger. 2013. "A Benchmark of Kriging-Based Infill Criteria for Noisy Optimization." *Struct. Multidiscip. Optim.* 48 (3): 607–626. https://doi.org/10.1007/s00158-013-0919-4.

Rasmussen, Carl Edward, and Christopher K. I. Williams. 2006. *Gaussian Processes for Machine Learning*. Cambridge, Mass: MIT Press.
